# Supplementary material for: Pleomorphic effects of three small-molecule inhibitors on transcription elongation by Mycobacterium tuberculosis RNA polymerase
Source: eLife. 2025 Oct 3;14:e105545. doi: 10.7554/eLife.105545 (PMC12558654; doi:10.7554/eLife.105545)
Supplement: Figure 3—source data 2. [file elife-105545-fig3-data2.zip › Figure 3 Source Data 2/Figure 3 Source Data 2.pdf]

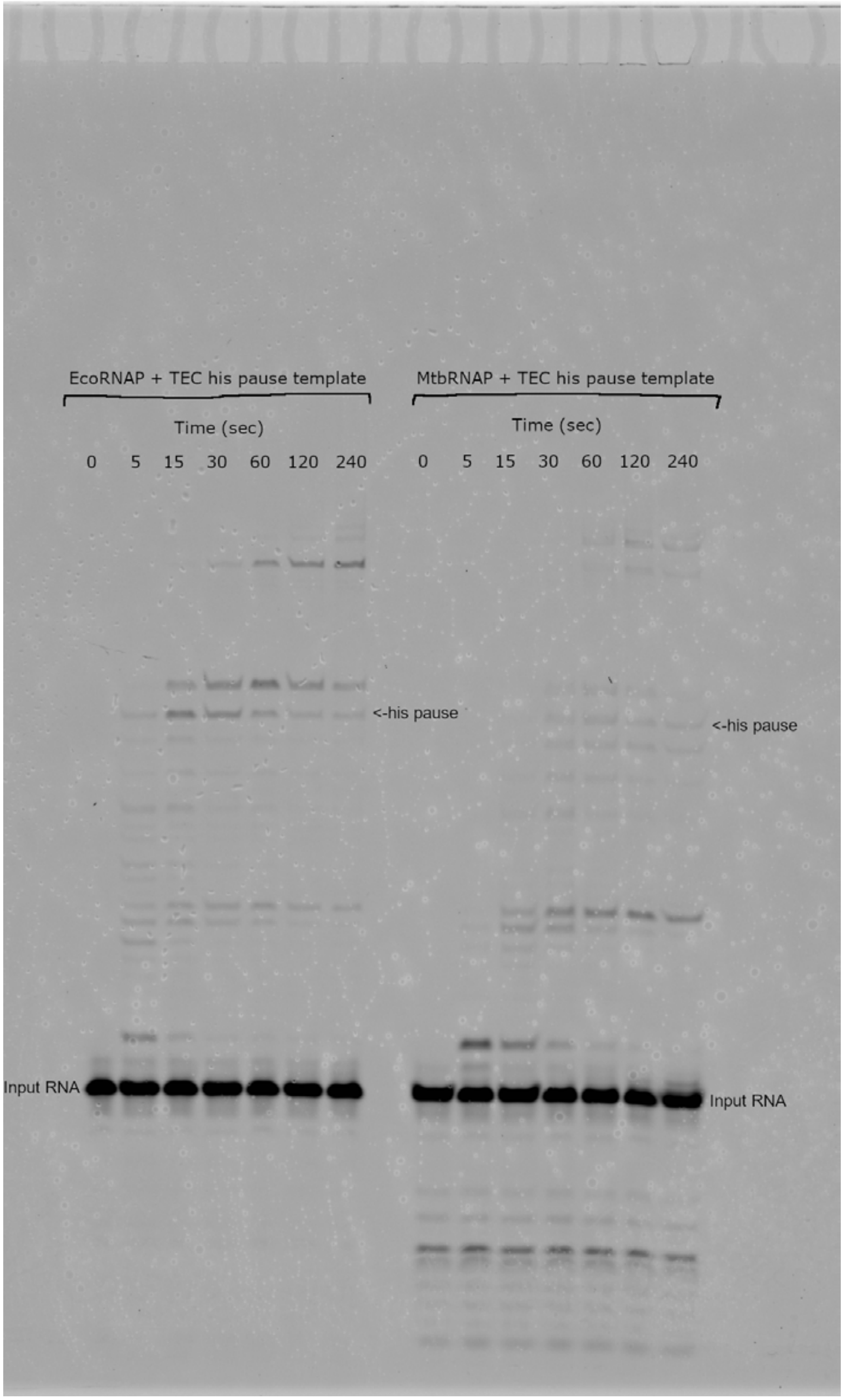
EcoRNAP + TEC his pause template
Time (sec)
0 5 15 30 60 120 240
MtbRNAP + TEC his pause template
Time (sec)
0 5 15 30 60 120 240
<-his pause
<-his pause
Input RNA
Input RNA

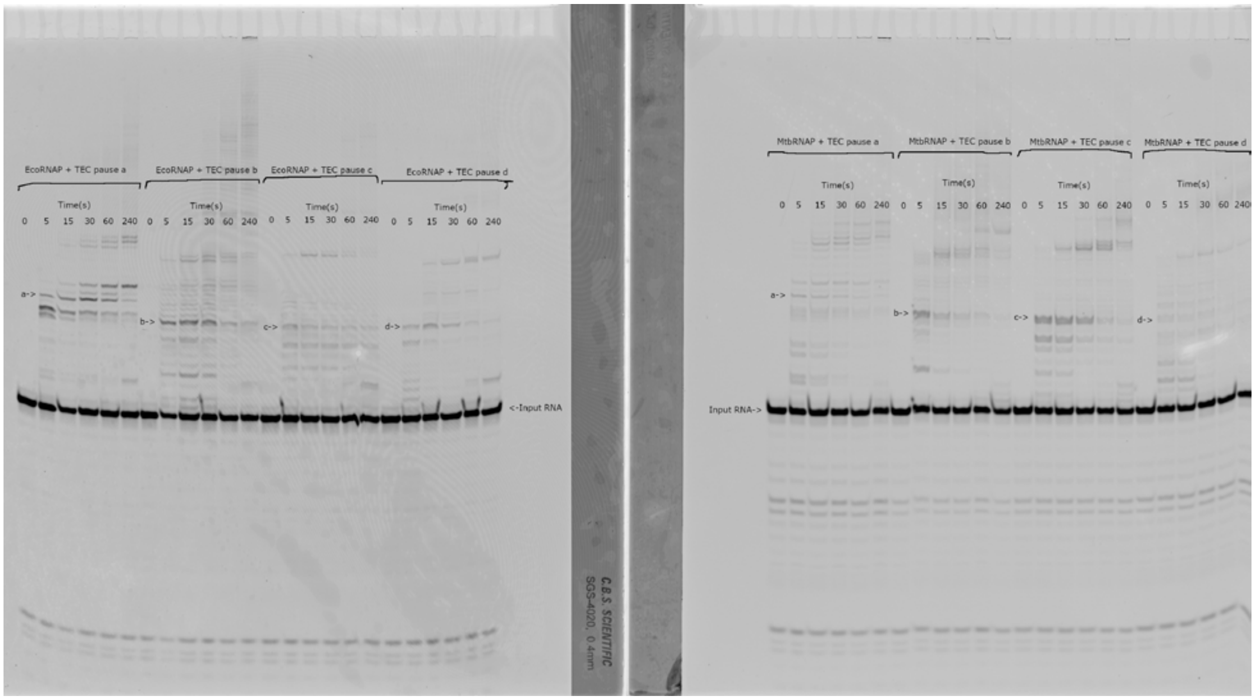

Figure 3- source data 2. Original RNA gel images for the time-course transcription experiments found in Figure 3d, with labels. This is a RNA elongation assay, where the FAM-labeled input RNA is extended by the polymerase over time. Timepoints refer to time after adding rNTPs. The first gel is for the his pause, the second pair of gels are for pauses a, b, c, and d.
