# Supplementary material for: Pleomorphic effects of three small-molecule inhibitors on transcription elongation by Mycobacterium tuberculosis RNA polymerase
Source: eLife. 2025 Oct 3;14:e105545. doi: 10.7554/eLife.105545 (PMC12558654; doi:10.7554/eLife.105545)
Supplement: Figure 3—figure supplement 1—source data 2. [file elife-105545-fig3-figsupp1-data2.zip › Figure 3-figure supplement 1-source data 2/Figure 3-figure supplement 1-source data 2.pdf]

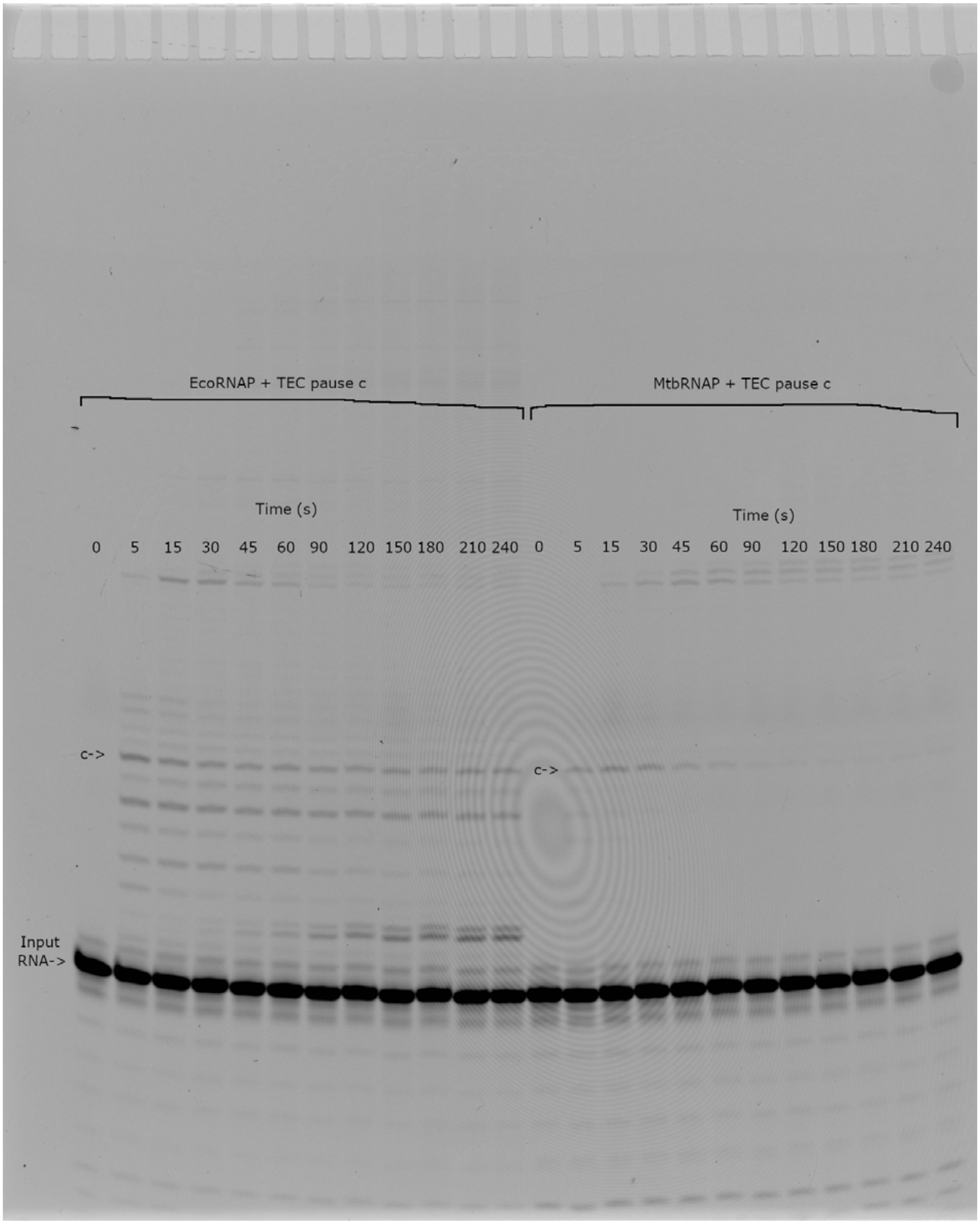

Figure 3-figure supplement 1-source data 2. Original RNA gel image for the time-course transcription experiments found in Figure 3-figure supplement 1, with labels. This is a RNA elongation assay, where the FAM-labeled input RNA is extended by the polymerase over time. Timepoints refer to time after adding rNTPs.
