# Supplementary material for: Pleomorphic effects of three small-molecule inhibitors on transcription elongation by Mycobacterium tuberculosis RNA polymerase
Source: eLife. 2025 Oct 3;14:e105545. doi: 10.7554/eLife.105545 (PMC12558654; doi:10.7554/eLife.105545)
Supplement: Supplementary file 1. [file elife-105545-supp1.docx]

Supplementary File 1: Tables of Oligos, Plasmids and Cells

Table of DNA Oligonucleotides

| **S/N** | **Sequences** | **Source** | **Identifier** | **Description** |
| --- | --- | --- | --- | --- |
| 1 | I2AmPr:  5′-ATGTCTCATCTGGCATTCG/i2AmPr/TCTCCTCTTAAACCTTCATCTGACAACGTG-3′ | IDT | Labstock: CBD411 | Template strand complementary to CBD410 for bubble assembly for measuring translocation rate. It forms a hybrid with RNA CBR14. |
| 2 | 5′-CACGTTGTCAGATGAAGGTTTAAGAGGAGATCGAATGCCAGATGAGACAT-3′ | IDT | Labstock: CBD410 | Non-template strand complementary to CBD411 for bubble assembly for measuring translocation rate. |
| 3 | 5’-CCTATAGGATACTTACAGCCATCGAGAGGCGGGTAGATCCGCCCGCATATCTCG-3’ | IDT | Labstock: NDS_A_54 | Non-template strand complementary to template strand TDS_A_54 for bubble assembly with RNA RNA7FL to form the consensus pause *a* transcription template. |
| 4 | 5’-CGAGATATGCGGGCGGATCTACCCGCCTCTCGATGGCTGTAAGTATCCTATAGG-3’ | IDT | Labstock: TDS_A_54 | Template strand complementary to NDS_A_54 for bubble assembly. |
| 5 | 5’-CCTATAGGATACTTACAGCCATCGAGAGGGGTGAAACCGCATCTTTTGACATCG-3’ | IDT | Labstock: NDS_B_54 | Non-template strand complementary to template strand TDS_B_54 for bubble assembly with RNA RNA7FL to form the consensus pause *b* transcription template. |
| 6 | 5’-CGATGTCAAAAGATGCGGTTTCACCCCTCTCGATGGCTGTAAGTATCCTATAGG-3’ | IDT | Labstock: TDS_B_54 | Template strand complementary to NDS_B_54 for bubble assembly. |
|  | 5′-CCTATAGGATACTTACAGCCATCGAGAGGGGTAAAGTGTACGTAGAAAAAGGCC-3′ | IDT | Labstock: NDS_C_54 | Non-template strand complementary to template strand TDS_C_54 for bubble assembly with RNA RNA7FL to form the consensus pause *c* transcription template. |
| 7 | 5′-GGCCTTTTTCTACGTACACTTTACCCCTCTCGATGGCTGTAAGTATCCTATAGG-3′ | IDT | Labstock: TDS_C_54 | Template strand complementary to NDS_C_54 for bubble assembly. |
| 8 | 5’-CCTATAGGATACTTACAGCCATCGAGAGGCGTATCACTGCGCGCCACATTCGCG-3’ | IDT | Labstock: NDS_D_54 | Non-template strand complementary to template strand TDS_D_54 for bubble assembly with RNA RNA7FL to form the consensus pause *d* transcription template. |
| 9 | 5’-CGCGAATGTGGCGCGCAGTGATACGCCTCTCGATGGCTGTAAGTATCCTATAGG-3’ | IDT | Labstock: TDS_D_54 | Template strand complementary to NDS_D_54 for bubble assembly. |
| 10 | 5’-CCTATAGGATACTTACAGCCATCGAGAGGCCTGACTAGTCTTTCAGGCGATGTGTGCTGGAAGACATTCAGATCGACCTGT-3’ | IDT | Labstock: NDS_81his | Non-template strand complementary to template strand TDS_81_his for bubble assembly with RNA RNA7FL to form the consensus *his* pause transcription template. |
| 11 | 5’-ACAGGTCGATCTGAATGTCTTCCAGCACACATCGCCTGAAAGACTAGTCAGGCCTCTCGATGGCTGTAAGTATCCTATAGG-3’ | IDT | Labstock: TDS_81his | Template strand complementary to NDS_81_his for bubble assembly. |
| 12 | p: phosphate  5'-pGTCGCGATAATCTGAATAACCATAATC-3' | IDT | Labstock: C_Avitag_Rev | Labeling at the C-terminal of rpoC the Mtb RNAP with Avitag from Plasmid pET DUET BC. |
| 13 | p: phosphate  5'-pATCGGCCTGAACGATATCTTCGAAGCGCAGAAAATTGAATGGCATGAATAGGTACCCTCGAGTCTGG-3' | IDT | Labstock: C_Avitag_Fdw | Labeling at the C-terminal of rpoC the Mtb RNAP with Avitag from Plasmid pET DUET BC. |
| 14 | p: phosphate  5'- pACAGGCGGTTAGGTACCCTCGAGTCTGGTAAAG-3' | IDT | Labstock: C_LPXTGX_Fdw | Labeling at the C-terminal of rpoC the Mtb RNAP with Avitag from plasmid pET DUET BC. |
| 15 | p: phosphate  5'- pTTCCGGTAAGTCGCGATAATCTGAATAACCATAATCATCCAG-3' | IDT | Labstock: C_LPXTGX_Rev | Labeling at the C-terminal of rpoC the Mtb RNAP with Avitag |
| 16 | 5’-GTTGCATGCCGACGGCCAGTG AATTCAAATATTT-3’ | IDT | Labstock: SphIAC50 | Amplify AC50 promoter + C-less 10bp cassette with SphI/ XbaI ends from pJET 1.2 AC50 to clone it into pUC19 |
| 17 | 5’-CGGGCCCGGGATCCTTCACTGCTC-3’ | IDT | Labstock: XbaIAC50 | Amplify AC50 promoter + C-less 10bp cassette with SphI/ XbaI ends from pJET 1.2 AC50 to clone it into pUC19 |
| 18 | 5’-TTTCTAGACCCACCTGGGTTGGTGTGCCC-3’ | IDT | Labstock: X27rpoCBB _for | Amplifying and cloning fragment of RpoBC genes of H37rv Mtb into pUC19 AC50. |
| 19 | 5’-GCCGGATCCGGTCTCGCGGTTCCACGCATCGCCCGGCTGCC-3’ | IDT | Labstock: X27rpoCBB_rev | Amplifying and cloning fragment of RpoBC genes of H37rv Mtb into pUC19 AC50. |
| 20 | p: phosphate  5’-pgccaagcttgcatgccgac-3’ | IDT | delLAcpUC19_for | Remove the LAC promoter from plasmid PUC19AC50 Mtb RNAP |
| 21 | p: phosphate  5’-pgcattaatgaatcggccaac-3’ | IDT | Labstock: delLAcpUC19_rev | Remove the LAC promoter from plasmid PUC19AC50 Mtb RNAP |
| 22 | 5’-gagatgcgccacaatgag-3’ | IDT | Labstock: WhiB1_fwd | Amplify DNA fragment containing synthetic WhiB1 terminator from whiB1_pUC57 plasmid. |
| 23 | 5’-acggccagtgaattcgag-3’ | IDT | Labstock: WhiB1_rev | Amplify DNA fragment containing synthetic WhiB1 terminator from whiB1_pUC57 plasmid. |
| 24 | p: phosphate  5’-paagaaagggaggaccatctctctagacccacctg-3’ | IDT | Labstock: gu29_fwd | Primer for round-the-horn mutagenesis to increase the cassette from 10 to 29 nt. Plasmid pSA4 -> plasmid pSGU29-A |
| 25 | p: phosphate  5’-pcttttctcttccttccctttaaatactagccgct-3’ | IDT | Labstock: gu29_rev | Primer for round-the-horn mutagenesis to increase the cassette from 10 to 29 nt. Plasmid pSA4 -> plasmid pSGU29-A |
| 26 | 5’-TACTTCCAATCCAATGCAATGACGGATACTCAAGTCACCTG-3’ | IDT | Labstock: 421_F | Amplification and LIC of MtbGreA gene on Plasmid pET_His6_TEV_LIC (lic1) Kanamycin r. |
| 27 | 5’-TTATCCACTTCCAATGTTATTCTAGGAGTGGTACGGCTCGG-3’ | IDT | Labstock: 421_R | Amplification and LIC of MtbGreA gene on Plasmid pET_His6_TEV_LIC (lic1) Kanamycin r. |
| 28 | 5’-AGTGCTGCAATGATACCGCG-3’ | IDT | Labstock: 1.5kbHandleBC_fdw | To amplify the 1.5 Kb Biotin Dig Handle from Plasmid pET-DUET-BC_avitag |
| 29 | 5’-Biotin-TCCATAGGCTCCGCCCCC-3’ | IDT | Labstock: 1.5kbHandleBC_rev | To amplify the 1.5 Kb Biotin Dig Handle from Plasmid pET-DUET-BC_avitag |
| 30 | AmMC6: Amino modifier with 6 carbon spacers  5’-AmMC6-TTAATTCATTGCGTTCTGTACACG-3’ | IDT | Labstock: amine_oligobead | Amine 5' oligo complementary to either 5´ACCG oligo or 5'CGGT oligo. For the preparation of Oligo Beads |
| 31 | p: phosphate  5’-pCGGTCGTGTACAGAACGCAATGAATT-3’ | IDT | Labstock: CGGT_oligobead | 5' oligo complementary to the amine oligo to form an overhand 5' CGGT oligo. For the preparation of Oligo Beads |
| 32 | p: phosphate  5’-pACCGCGTGTACAGAACGCAATGAATT-3’ | IDT | Labstock: ACCG_oligobead | 5' oligo complementary to the amine oligo to form an overhand 5' ACCG oligo. For the preparation of Oligo Beads |
| 33 | 5’-catgccgacggccagtgaattc-3’ | IDT | Labstock: AC50_rBC_for | Amplify a DNA template (~2.9 Kb) for opposing mode from plasmid pUC19_AC50_Mtb_rpoBC |
| 34 | Dig: Digoxygenin  5’-Dig-tgaattcgagctcggtacccgg-3’ | IDT | Labstock: DIg_AC50_rBC_rev | Amplify a DNA template (~2.9 Kb) for opposing mode from plasmid pUC19_AC50_Mtb_rpoBC |
| 35 | Dig: Digoxygenin  5’-Dig-gggtctcgcggtatcattgcag-3’ | IDT | Labstock: Dig_AssistBC_For | Amplify a DNA template (~4 Kb) for assisting mode from plasmid pUC19_AC50_Mtb_rpoBC |
| 36 | 5’-tgaattcgagctcggtacccgg-3’ | IDT | Labstock: AssistBC_rev | Amplify a DNA template (~4 Kb) for assisting mode from plasmid pUC19_AC50_Mtb_rpoBC |
| 37 | 5’-Biotin-ATTCGTGGAGGCCGTCGG-3’ | IDT | Labstock: CBD 316 (biotin forward) | Amplify a 1.5 Kb DNA handle modified in one end with biotin and in the other with two digoxigenins using PCR from Lambda Genome. |
| 38 | Dig: Digoxygenin  5'-Dig-CGGGAGTGATTTCCGTCT(Dig)ACGGT-3' | IDT | Labstock: Internal Dig (Double Dig Primer) Rev | Amplify a 1.5 Kb DNA handle modified in one end with biotin and in the other with two digoxigenins using PCR from Lambda Genome. |

Table of RNA Oligonucleotides

| **S/N** | **Sequences** | **Source** | **Identifier** | **Descriptions** |
| --- | --- | --- | --- | --- |
| 1 | FAM: Fluorescein  5’-FAM-CACUAACUAAGAGGAG-3’ | IDT | Labstock: CBR14 | RNA to assemble a transcription bubble for the real time measurement of the transcription translocation rate. |
| 2 | FAM: Fluorescein  5’-FAM-UUCAUUCCCGAGAGG-3’ | IDT | Labstock: RNA7FL | RNA to assemble transcription bubbles with DNA template and non-template strand of the consensus pauses *a*, *b*, *c,* *d*, and *his*. |

Table of Plasmids, DNA templates, and Genomic DNA

| **S/N** | **Source** | **Identifier** | **Description** |
| --- | --- | --- | --- |
| 1 | Herrera-Asmat *et al.* 2017^60^ | pACYC-DUET-AOZ | Co-expression of alpha, omega, and sigma A subunits of MtbRNAP in *E. coli.* |
| 2 | This work | pET-DUET-BC-avitag | Co-expression of beta and avitagged- beta prime subunits of MtbRNAP in *E. coli.* |
| 3 | Professor Nicola Burgess-Brown | pCDF-BirA | Co-expression of BirA for in vivo biotinylation of avitagged-beta prime subunit of MtbRNAP in *E. coli.* |
| 4 | This work | pET-DUET-BC-sortag | Co-expression of beta and sortagged-beta prime subunits of MtbRNAP in *E. coli.* |
| 5 | Addgene, # 29653 | pET His6 TEV | Expression and LIC Plasmid for Mtb transcription factors. |
| 6 | NEB | pUC19 | Multicloning plasmid |
| 7 | GenScript | pJET 1.2 AC50 | Plasmid harboring synthetic AC50 promoter + 10 stall cassette. |
| 8 | This work | pUC19-AC50 | pUC19 plasmid harboring AC50 promoter + 10 stall cassette. |
| 9 | This work | pUC19_AC50_Mtb_rpoBC | pUC19-AC50 plasmid harboring a ~2.7kb fragment of rpoB and rpoC genes from *M. tuberculosis* Erdman. |
| 10 | This work | pUC19-del-LAC Ac50 MtbRpoBC | Plasmid pUC19_AC50_Mtb_rpoBC with the Lac promoter deleted. |
| 11 | This work | pSA4 | pUC19-del-LAC Ac50 MtbRpoBC with ~ 1.5 Kb deleted from rpoC gene with Eco53kI |
| 12 | This work | pSGU29-A | pSA4 plasmid with mutation extending the stalling Cassette |
| 13 | Genscript | whiB1_pUC57 | Plasmid harboring a synthetic WhiB1 terminator sequence. |
| 14 | Professor Sara Stanley | Genomic DNA from *M. tuberculosis* Erdman | Source for *M. tuberculosis* genes as a template of PCR during molecular cloning. |
| 15 | This work | pSGU_W | pSGU29-A plasmid with the cloned terminator WhiB1 by Hifi assembly |
| 16 | This work | pSGUML1T1 | pSGU_W plasmid with the cloned Mtb pause eight repeated by Hifi assembly. |
| 17 | Herrera-Asmat *et al.* 2017^60^ | pET-his-tev-MtbCarD (8PC) | Plasmid for expression of recombinant MtbCarD in *E. coli*. |
| 18 | This work | pET His6 TEV GreA (Rv1080c) | Plasmid for expression of recombinant MtbGreA in *E. coli*. |
| 19 | Gabizon *et al.* 2018^13^ | ddATP pGMZ-His T7A | ~3.4 kbp DNA template containing the 8 repeats in tandem of consensus *a, b, c, d,* and *his* pauses from *E. coli* for assisting force in EcoRNAP. |
| 20 | This work | ddCTP_pSGUML1T1 | ~2.2 kbp DNA template containing 8 repeats in tandem of *c* pause from *E. coli* for opposing force in MtbRNAP |
| 21 | This work | ddATP_pSGUML1T1 | ~ 2.2 kbp DNA template containing 8 repeats in tandem of *c* pause from *E. coli* for assisting force in MtbRNAP |
| 22 | This work | OF 2.9Kb AC50MtbRpoBC | ~2.9 kbp DNA template of AC50 promoter and a fragment of Mtb rpoB and rpoC genes for opposing force in MtbRNAP |
| 23 | This work | AF 4Kb AC50MtbRpoBC | ~ 4.0 kbp DNA template of AC50 promoter and a fragment of Mtb rpoB and rpoC genes for assisting force in MtbRNAP. |
| 24 | This work | 1.5 Kb Biotin Dig Handle | ~1.5 kbp DNA handle with one biotin in one end and a double dig in the other. |
| 25 | This work | 1.5 Kb ACCG Handle | ~1.5 kbp DNA handle with one biotin in one end and an overhand with 5’P-ACCG. |

Table of Competent Cells

| **S/N** | **Source** | **Identifier** | **Usage** |
| --- | --- | --- | --- |
| 1 | Thermo Scientific | Invitrogen™ One Shot™ Mach1™ T1 Phage-Resistant Chemically Competent E. coli. Catalog #C862003 | These cells were used for the creation and amplification of all plasmids except for those containing a molecular ruler. |
| 2 | New England Biolabs | NEB® Stable Competent E. coli (High Efficiency). Catalog #C3040H | These cells were used for the creation and amplification of the plasmids containing a molecular ruler. |
| 3 | Thermo Scientific | BL21(DE3) Competent Cells. Catalog #EC0114. | These cells were used for expression of all proteins. |
